# Imaging studies of bacterial biofilms on cochlear implants—Bioactive glass (BAG) inhibits mature biofilm

**DOI:** 10.1371/journal.pone.0229198

**Published:** 2020-02-21

**Authors:** Lisa Kirchhoff, Diana Arweiler-Harbeck, Judith Arnolds, Timon Hussain, Stefan Hansen, Ralph Bertram, Jan Buer, Stephan Lang, Joerg Steinmann, Benedikt Höing

**Affiliations:** 1 Institute of Medical Microbiology, University Hospital Essen, University Duisburg-Essen, Essen, Germany; 2 Department of Otorhinolaryngology, Head and Neck Surgery, University Hospital Essen, University Duisburg-Essen, Essen, Germany; 3 Institute of Clinical Hygiene, Medical Microbiology and Clinical Infectiology, Paracelsus Medical University, Klinikum Nuernberg, Nuremberg, Germany; VIT University, INDIA

## Abstract

The capability of *Pseudomonas aeruginosa* and *Staphylococcus aureus* to form biofilm on varying CI component materials differs in the presence and absence of bioactive glass (BAG). The application of BAG induces significant changes in biofilm morphology which can be visualized via scanning electron microscopy (SEM). Bacterial biofilm formation on medical devices, such as cochlear implants (CI), can lead to chronic infections. Interestingly, BAG of type S53P4 seems to be a promising tool for use in the reduction of biofilm development. Primarily, four bacterial species known to cause implant-related infections, *P*.*aeruginosa* (ATCC9027), *S*. *aureus* (ATCC6538), *Staphylococcus epidermidis* (ATCC12228) and *Streptococcus pyogenes* (ATCC19615) were analyzed regarding their capacity to form biofilm on CI components manufactured from three kinds of material: silicone, platinum and titanium. Subsequently, *P*. *aeruginosa* and *S*. *aureus* biofilms were visualized using scanning electron microscopy, comparing BAG-treated biofilm with non-treated biofilm. The four bacterial species presented biofilm-forming capabilities in a species and surface dependent manner. Metal CI components allowed for the greatest proliferation of biofilm. *S*. *aureus* and *P*. *aeruginosa* showed the highest rate of biofilm formation on polystyrene surfaces. For both species, SEM revealed altered biofilm morphology after treatment of S53P4 BAG. This study indicates that bacterial biofilm formation and structure on CI components is dependent on the surface composition, altering between metal and silicone surfaces. After application of BAG, changes in biofilm morphology on CI components were observed. These data highlight the impact of BAG on bacterial biofilm morphology.

## Introduction

In cochlear implants (CIs), sound is transduced via an externally located microphone to internal parts of the implant, which are located under the temporal skin. A surgically inserted electrode located in the cochlea directly stimulates the hair cells in the organ of Corti in a frequency-dependent manner [1]. Implanting exogenous material such as prostheses or technical devices in a human body is often associated with complications such as perioperative infections. Although these infections are reported to be complications with low incidence in cochlear implant surgery [2,3], the clinical implications such as extended length of stay and higher morbidity are substantial. In this setting, high costs of a surgical implant replacement and the mandatory implant restriction time for the patient as significant socioeconomic and individual patient-related consequences must be considered. The main reason for implant-related infections is the presence of bacteria, being able to adhere to abiotic surfaces, forming biofilms on parts of the implant. In a biofilm, microorganisms are embedded in an extracellular matrix (ECM), leading to resistance against immune cells and antibiotic therapy [4,5]. These biomaterial-associated infections are frequently caused by staphylococci (*Staphylococcus aureus* and *Staphylococcus epidermidis)*, by streptococci, e.g. *Streptococcus pyogenes* as well as by Gram-negative bacteria such as *Pseudomonas aeruginosa* [6].

Consequently, prevention of device-associated infections from biofilm-forming bacteria are of high scientific and clinical interest. Thereby, synthetic bioactive glass (BAG) emerged as a promising tool. BAG of type S53P4 consists of silicon dioxide, sodium oxide, calcium oxide and phosphorus pentoxide [7]. Its osteoinductive [8] and antibacterial properties have been demonstrated, e.g. against the microbiota of human oral cavities [9]. The antibiofilm activity can be determined by crystal violet staining and confocal laser scanning microscopy (CLSM) assays [10]. Furthermore, S53P4 has proven its efficacy in biofilm prevention on prosthesis materials such as middle ear prostheses and in fracture fixtures [10,11]. A recent investigation demonstrated biofilm reduction after application of BAG to CI components [12].

However, only a small number of studies have been undertaken focusing on visualization of bacterial biofilms on CIs using electron microscopy [13,14]. The CI device itself, which consists of diverse materials (silicone vs. metal) has also not been sufficiently considered so far. Moreover, bilateral interactions between BAG and the surface structure of CI components have yet to be analyzed. In summary, there is a lack of evidence concerning visualization of bacterial biofilm on CI components and potential interactions with BAG. Therefore, the aim of this investigation was to identify the surface-specific bacterial preferences and to understand biology, morphology and potential surface—bacteria interactions of a biofilm forming matrix on implant materials, leading eventually to the development of clinical tools to reduce the risk of peri- and postoperative infections.

In this study, four bacterial species frequently causing biomaterial-associated infections, *S*. *pyogenes*, *S*. *aureus*, *S*. *epidermidis* and *P*. *aeruginosa* were analyzed for biofilm formation capability in the preliminary analysis. The strains with the strongest biofilm formation were used for imaging and application of bioactive glass (BonAlive^®^ Biomaterials LTD, Turku, Finland). Biofilm formation was visualized via scanning electron microscopy (SEM).

## Material and methods

### Strains

Four different bacterial species were used for the evaluation of biofilm formation on CI components: *S*. *pyogenes* (ATCC 19615), *P*. *aeruginosa* (ATCC 9027), *S*. *aureus* (ATCC 6538) and *S*. *epidermidis* (ATCC 12228). For the image-based investigation of anti-biofilm effects, *S*. *aureus* and *P*. *aeruginosa* were chosen as common bacterial strains responsible for biofilm-related implant infections.

### Cochlear implant compounds

The commercially available implant kits from three different suppliers of CIs were used in this study: The Hi Res 90K^™^ implantable cochlear stimulator (Allergy Kit, 7095653–001) from ADVANCED BIONICS LLC (Valencia, California, USA), SYNCHRONY (Material sample, D9000) from MED-EL (Innsbruck, Austria) and Nucleus^®^ CI512 (Material sample kit Z290707) from Cochlear^™^ (Sydney, Australia). All implant kits were composed of various silicone rubber, platinum and titanium parts. The sample pieces used in this study are the components, which are directly in contact with the patient’s body when implanted. For each species, one silicone, titanium and platinum sample piece were used to investigate the surface for biofilm formation and imaging.

### Cultivation and biofilm formation

Strains were cultured on Columbia agar containing sheep blood at 36°C overnight. One colony was selected and transferred to tryptic soy broth (TSB) for further cultivation at 36°C under gentle agitation (120 rpm) overnight. Biofilm cultivation was carried out as previously described [12]. Briefly, cultures were washed in phosphate buffered saline (PBS) and diluted 1:200 in TSB, which equals a cell concentration between 5 x 10^5^ and 5 x 10^6^ cells/mL for each strain. Each of the sterile implant components was placed in a well of a sterile, polystyrene, 6-well microtiter plate and the cell suspension was added to the well covering the CI component. As a control, biofilm was allowed to form on the polystyrene surface of one well of the plate. As a negative control, sterile media without bacterial inoculation was added to another well.

Biofilm formation on polystyrene was quantified by crystal violet (0.1%) staining for 20 min at room temperature after washing with PBS. Repeated washing was followed by destaining of the overnight-dried wells using 30% acetic acid. Optical density measurements were performed at a wavelength of 620 nm and values were corrected by background reading.

### Scanning electron microscopy (SEM)

Biofilms were cultivated on the implant surfaces as described above and washed with PBS: for scanning electron microscopy (SEM), the implants with the biofilm were coated with 5 nm thick platinum using high resolution ion beam coating (gatan model 681). FEI Quanta 400 SEM (TSS Microscopy, Hillsboro, USA) was used for imaging. Images were taken at various magnifications.

### Antibiofilm effect of BAG granules

The effect of BAG granules (S53P4, BonAlive^®^; BonAlive Biomaterials LTD, Turku, Finland) on *S*. *aureus* and *P*. *aeruginosa* biofilm morphology on CI surfaces was analyzed by SEM. Biofilm was cultivated on the CI components as described above for a period of 24 hours. Non-adherent cells were removed by washing with PBS and glass granules were added to the surface and incubated for another 24 hours at 36°C. As a control, sterile fresh media without BAG granules was utilized. Imaging was processed as described above.

### Image analysis

The SEM images were analyzed using ImageJ (Wayne Rasband. Rasband, W.S., ImageJ, U. S. National Institutes of Health, Bethesda, Maryland, USA,). Cell sizes were measured as well as gray-value means. Statistical analysis was performed using GraphPad Prism6 (GraphPad Software Inc., La Jolla, CA, USA). Statistical differences were analysed using an unpaired T-test.

## Results and discussion

Firstly, the biomass in biofilm of the common bacterial pathogens *S*. *aureus*, *S*. *epidermidis*, *S*. *pyogenes* and *P*. *aeruginosa* formed in the polystyrene wells was determined in a crystal violet stain assay (Table 1).

**Table 1:** Optical density corrected data at 620 nm of bacterial (*S*. *aureus* (ATCC 6538), *S*. *epidermidis* (ATCC 12228), *S*. *pyogenes* (ATCC 19615) and *P*. *aeruginosa* (ATCC 9027)) biofilms.

	*S*. *aureus*	*S*. *epidermidis*	*S*. *pyogenes*	*P*. *aeruginosa*
1	2.9	0.725	1.36	3.83
2	4.78	0.725	1.82	2.71
3	3.9	0.59	1.35	2.05

Biofilms were formed on polystyrene surface for 24 h at 36°C, washed thrice with PBS and stained with 0.1% crystal violet for 20 minutes. After repeated washing, biofilms were air dried in the dark and destained via incubation with 30% acetic acid. Solutions optical density at a wavelength of 620 nm was read in a microplate reader (Tecan Sunrise^™^, Tecan Trading AG, Switzerland). Background readings were subtracted.

**Fig 1:**
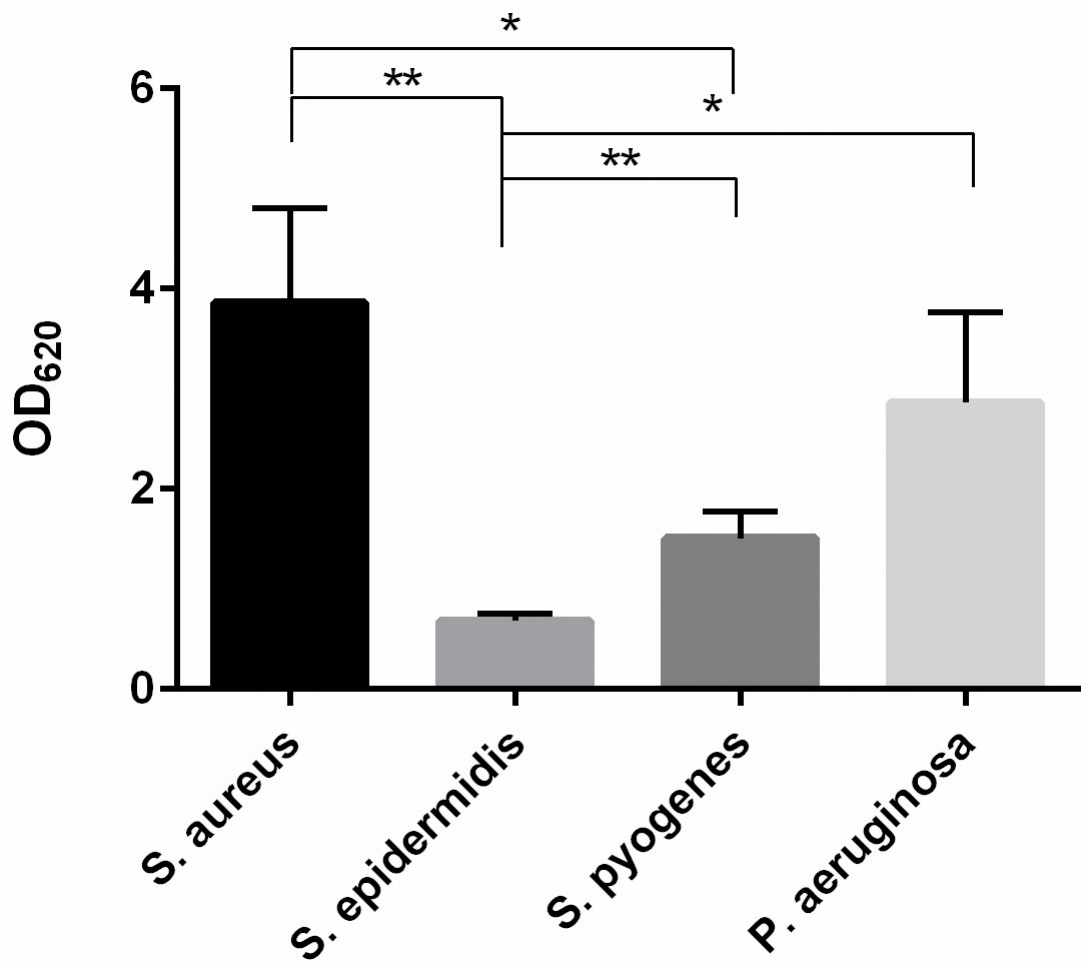
Biomass in biofilm of *S*. *aureus* (ATCC 6538), *S*. *epidermidis* (ATCC 12228), *S*. *pyogenes* (ATCC 19615) and *P*. *aeruginosa* (ATCC 9027) on polystyrene. *: p < 0.05, **: p < 0.01, n = 3.

While *S*. *epidermidis* exhibited the lowest biofilm formation, *S*. *aureus* and *P*. *aeruginosa* exhibited the highest quantity of biomass in biofilm as detected by optical density reading (Fig 1). Thus, the latter were further used in microscopy experiments.

**Fig 2:**
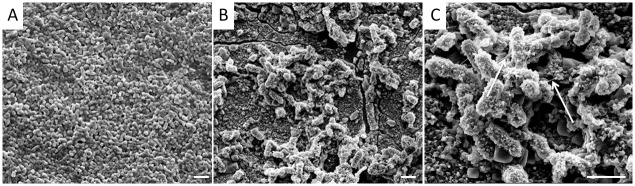
*P*. *aeruginosa* biofilm on platinum (part of Hi Res 90K^™^ implantable cochlear stimulator, Advanced bionics), formed during 24 h at 36°C. A: After washing, incubated with fresh medium for a further 24 h. B & C: Additionally treated with S53P4 and incubated for a further 24 h. Rulers indicate a length of 2 μm. Magnifications are 10,000-fold in A and B and 25,000-fold in C. Arrows in C hint towards the protein dominated viscous appearing structures.

In order to visualize the biofilms, electron microscopy was utilized. The SEM images of *P*. *aeruginosa* biofilm on platinum showed rod-shaped cells with a length of approximately 2 μm, embedded in extracellular polymeric substances (EPS) (Fig 2A). The cells formed complex clusters (Fig 2B). The S53P4-treated biofilm showed cell surfaces, which were covered by a protein-rich structure, viscous in appearance (arrows, Fig 2C). When comparing treated biofilms with the control, the cell cluster in the treated sample was weaker with lower EPS formation. Gaps in the mature *P*. *aeruginosa* biofilm were observed after S53P4 treatment (Fig 2C).

**Fig 3:**
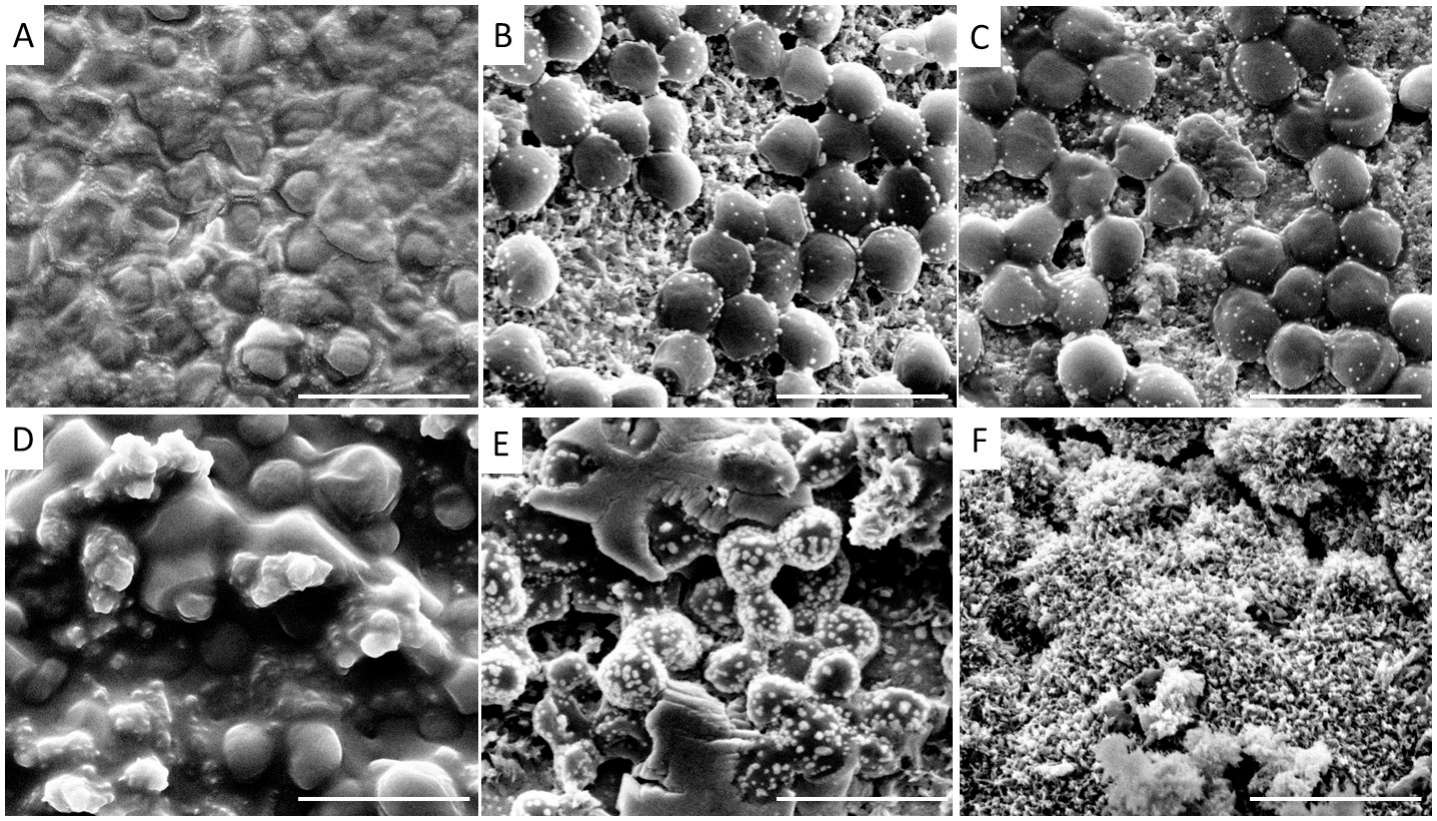
*S*. *aureus* biofilm visualized by scanning electron microscopy. A, D: on silicone; B, E: on platinum; C, F: on titanium. Biofilm was incubated with fresh media (A-C) or media with S53P4 (D-F). Rulers indicate a length of 2 μm. Magnifications are 50,000-fold.

SEM of *S*. *aureus* biofilm showed a dense cluster of cocci embedded in a matrix of extracellular material (Fig 3). A high morphological diversity of biofilm structure was detected in a surface material-dependent manner. The EPS matrix appeared densest on silicone (Fig 3A). The treatment with S53P4 resulted in the loss of a huge amount of EPS and loss of biofilm density (Fig 3D–3F). On platinum, the cells were mainly characterized by an altered morphology. The cell surface was covered with structures that are pale in appearance. These structures are larger and present in higher amounts on the surface of the S53P4 treated sample (Fig 3E) compared to the non-treated control. These structures were also observed on the cell surfaces of *S*. *aureus* on titanium (Fig 3C), but not detected on the surfaces of cells within the biofilms formed on silicone. In the treated biofilm, no cells were detected (Fig 3F).

**Fig 4:**
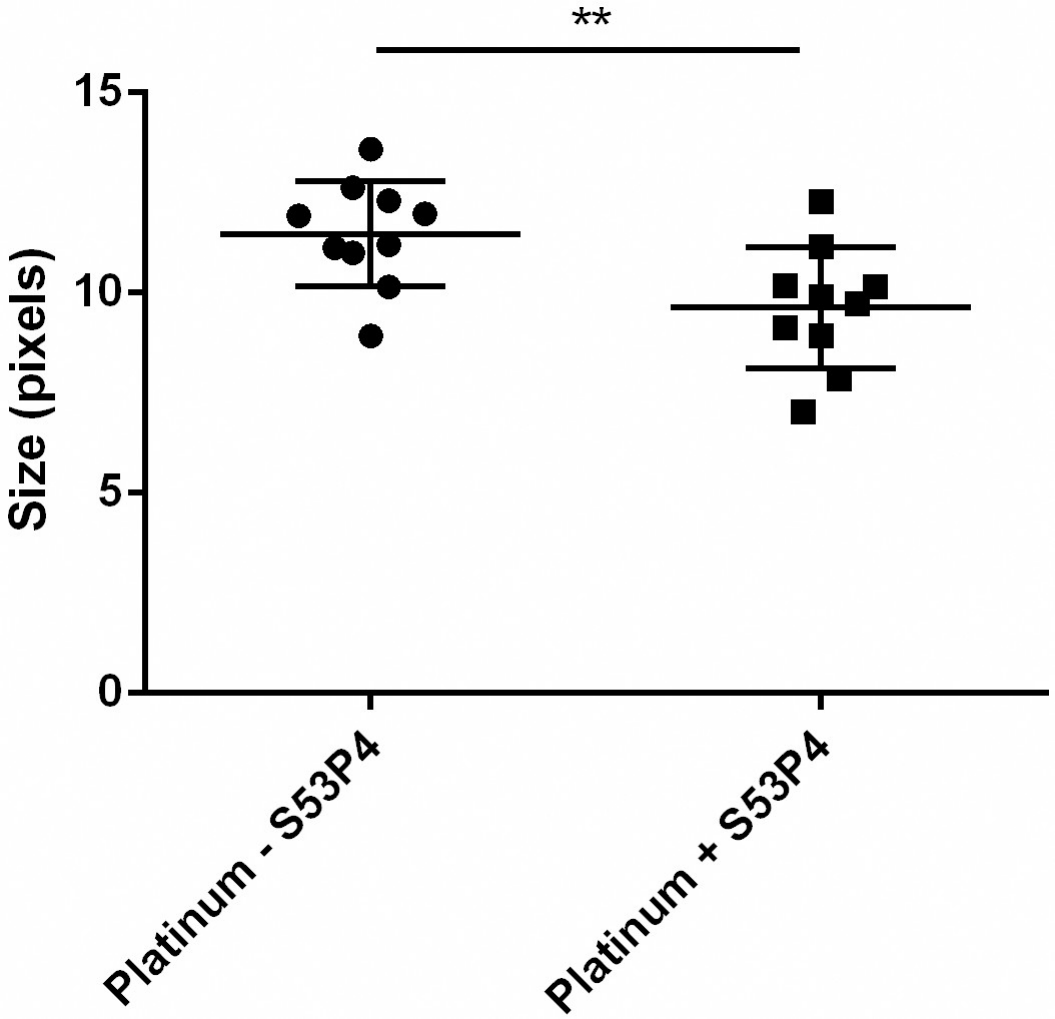
Mean gray scale of *S*. *aureus* biofilm cells on platinum with (+ S53P4) or without BAG (- S53P4) as determined by SEM image analysis using ImageJ. **: p < 0.01. n = 10.

These pale structures were significantly more abundant on the cells within the S53P4-treated biofilm formed on platinum, compared to the control (Fig 3B and 3E). This significant difference was confirmed by gray scale measurements using ImageJ (Table 2, Fig 4). A high gray scale value implies lighter colors, low values dark colors. The mean gray scale value for non-treated biofilm was 85.1 and for the treated biofilm a gray scale value was 138.7.

**Table 2:** Gray scale data of *S*. *aureus* biofilm cells on platinum with (+S53P4) or without BAG (- S53P4) as determined by SEM image analysis using ImageJ.

	Platinum - S53P4	Platinum + S53P4
1	98.955	143.738
2	58.426	117.874
3	102.201	114.355
4	79.276	165.787
5	120.858	132.027
6	97.379	147.696
7	108.926	127.211
8	13.4899	142.082
9	50.668	129.443
10	121.224	167.262

**Fig 5:**
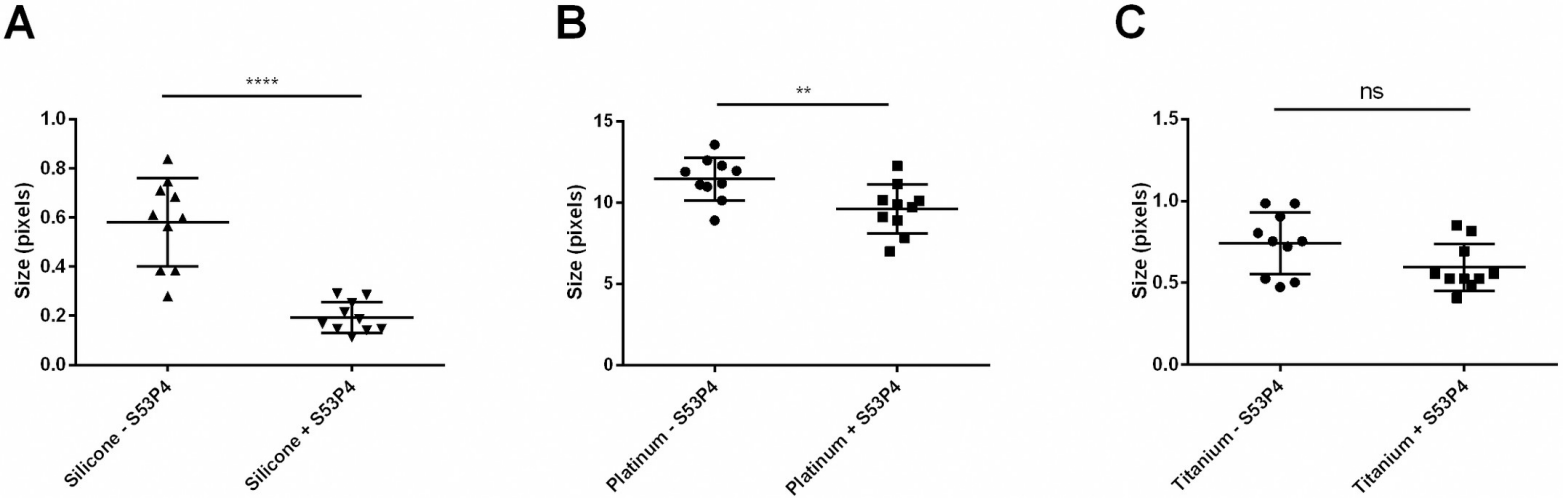
Relative cell sizes of *S*. *aureus* biofilm cells on platinum (A), titanium (B) and silicone (C) as determined by SEM image analysis using ImageJ. Ns = not significant. ****: p < 0.0001, **: p < 0.01. n = 10.

Furthermore, cell-sizes were ascertained to vary between the treated and the control biofilms. The cells of the non-treated biofilm were significantly larger compared to the ones treated with S53P4 when biofilms were formed on platinum or silicone. In addition, *S*. *aureus* cell sizes were smaller on titanium and silicone than on platinum (Fig 5, Table 3).

**Table 3:** Relative cell sizes of *S*. *aureus* biofilm cells on platinum, titanium and silicone as determined by SEM image analysis using ImageJ.

	Platinum - S53P4	Platinum + S53P4	Silicone - S53P4	Silicone + S53P4	Titanium - S53P4	Titanium + S53P4
1	8.901	10.126	0.385	0.114	0.755	0.486
2	13.573	12.256	0.711	0.186	0.503	0.556
3	11.181	7.003	0.611	0.167	0.905	0.556
4	11.951	9.877	0.748	0.251	0.475	0.526
5	11.108	9.106	0.566	0.14	0.804	0.526
6	10.976	11.14	0.28	0.285	0.526	0.85
7	12.276	9.71	0.685	0.291	0.722	0.817
8	10.133	10.169	0.839	0.214	0.755	0.694
9	12.604	8.902	0.385	0.146	0.986	0.408
10	11.908	7.843	0.599	0.146	0.984	0.525

Device-associated infections with biofilm forming bacteria pose a considerable risk for patients. Therefore, the inhibition of bacterial biofilm formation on medical devices, including cochlear implants is of high scientific interest. BAG has previously been shown to exhibit anti-biofilm activity [9,10]. In this study, the anti-biofilm activity of BAG granules was visualized by electron microscopy. Along with reduction of biofilm density upon exposure to S53P4, morphological changes of the ECM and cells within the cell cluster were detected.

Bacterial colonization of implants has been shown to occur on infected CIs and CIs in healthy individuals [15]. The overall incidence of post-operative infections in cochlear implant surgeries was reported to be approximately 4% [3]. Among others, one important risk factor for infection after CI surgery is the history of chronic ear diseases [3]. These post-operative infections are frequently caused by biofilm forming microorganisms associated with high resistance rates against anti-infective therapy [4]. Prominent colonizers of CIs and a cause of device-related infections are *S*. *aureus*, *S*. *epidermidis*, *S*. *pyogenes* and *P*. *aeruginosa*. In a standard biofilm formation assay, all species showed biofilm formation in a species and surface dependent manner [12]. Increased biomass in biofilm was measured on metal surfaces compared to that of biofilms formed on silicone, even if this difference was not deemed statistically significant [12]. In this analysis, the highest mass of biofilm was formed by *S*. *aureus*, followed by *P*. *aeruginosa*. Thus, these two species were selected for biofilm imaging studies using BAG.

BAGs are currently used in clinics and have emerged in several application fields, e.g. bone graft substitution. It has also been shown that BAG acts as an anti-infective agent, e.g. against *S*. *aureus* in chronic osteomyelitis when BAG granules were applied as a bone substitute [16]. Thereby, BAG also exhibited osteoconductive properties, vascular stimulation and antibacterial effects [17]. Additional to its proven effects of bacterial growth inhibition, BAG granules have recently been reported to inhibit mature bacterial biofilm on CI components [12]. Here, this anti-biofilm effect was visualized for the first-time using SEM. The SEM data revealed a less dense cell cluster as well as lower density ECM after treatment with S53P4.

Shifting environmental parameters to unfavorable conditions usually elicits increased rates of stress in bacterial cells. BAG granules are known to induce oxidative stress and to increase osmotic pressure [9,18]. Oxidative stress caused by BAG has been shown to inhibit fungal growth, biofilm formation as well as dimorphism of the yeast *Candida albicans* [19]. Growth inhibiting effects have also been detected against *S*. *aureus* and *P*. *aeruginosa* [20]. A comparable effect of ferric oxide and chitosan-coated silver nanoparticles against various bacteria, e.g. *S*. *aureus* and *P*. *aeruginosa*, has been reported [21,22]. S53P4 granules are described as releasing sodium from its surface after contact with body fluids, thereby increasing the pH into the alkaline range. Concurrently, osmotic pressure is increased due to the release of sodium, silicon, calcium, and phosphorus ions [9]. Both effects have been observed to contribute to anti-infective activities [23]. Growth inhibiting effects have been detected against more than 29 clinically relevant bacteria, including *P*. *aeruginosa* and *S*. *aureus* [24,25]. Also morphological changes, e.g. cell deformation and poration of cell membranes have been detected in methicillin-resistant *S*. *aureus* when brought into contact with BAG [18,26]. Accordingly, cell deformation of the BAG-treated *S*. *aureus* biofilm cells was detected in this study, which may represent a bacterial response to external osmotic pressure.

Another important finding was the reduction in density of the extracellular matrix after the application of BAG. The loss of ECM coincided with an altered morphology of extracellular substances. Viscous structures were observed in high amounts in the treated *P*. *aeruginosa* biofilm, whereas the non-treated control did not exhibit viscous-looking structures within the cell cluster. This phenomenon might indicate altered protein secretion dependent on stress initiated by BAG. The formation of small colony variant (SCV) phenotypes of staphylococci in stress or chronic conditions, e.g. antibiotic treatment, is a well-known phenomenon [27] and is accompanied by several morphological changes. This morphological alteration includes a more prevalent ECM as previously reported for *S*. *aureus* when treated with vancomycin [28]. A decrease in cell-size has also been detected within the BAG-treated biofilms. As previously described, it is known that S53P4 influences osmotic pressure. This may lead to the altered cell sizes. Stress application in various forms has been shown to result in cell-size loss in the manner of SCVs in *S*. *aureus* [28]. In conclusion, BAG granules demonstrated comparable effects on biofilm to those of various nanoparticles previously described in the cited literature [19,20]. The similarity in effect of these substances may correlate to their ability to induce cellular stress.

The appearance of pale structures on *S*. *aureus* surface occurred solely on metal surfaces and was increased in the treated samples compared to the non-treated control. One possible explanation could be that the bright structures on cell surfaces represent metal particles that were detached from the CIs and adhered to the bacterial surfaces. The adherence may be increased due to cell-surface charges when BAG was added. This notion is supported by lower gray-values detected in treated compared to non-treated samples.

## Conclusions

This investigation provides novel data concerning *S*. *aureus* and *P*. *aeruginosa* biofilm morphology on distinct cochlear implant components. After the application of BAG-S53P4 (BonAlive^®^) significant alterations in biofilm morphology could be detected via SEM. These changes in morphology include the cell size as well as the cell-surface appearance as detected for *S*. *aureus* and a less prevalent extracellular matrix for *P*. *aeruginosa*.
